# Legal Notes for Institutional Workers

**Published:** 1919-03-08

**Authors:** 


### LEGAL NOTES FOR INSTITUTIONAL WORKERS.

Houses used as Hospitals.—The case of *Frost* v. *King Edward VII. Welsh National Memorial for Prevention, etc., of Tuberculosis* (72 J. P. Rep., 249), the full official report of which is now available, will remain a leading case on two important questions relating to hospitals. First, as to how the erection of them in a district is affected by the general law of nuisance; and secondly, as to how the usual covenants in a ground lease prohibiting "noisy, noisome, or offensive businesses" stand in relation to hospital buildings. Though the plaintiffs were, on other stipulations in the lease, held entitled to an injunction, the case is an authority for saying that neither is a hospital a nuisance at common law, nor is the fact of carrying it on upon premises subject to leasehold covenants of the kind referred to a breach of such covenants. In the words of Mr. Justice Eve: "To hold that the carrying on of such an institution must involve a breach of that part of the covenant would, I think, be enlarging the operation of such a covenant beyond reasonable limits. I am not saying that there may not be many persons to whom the proximity of pain and suffering and the obtrusion before their eyes of its victims may not be annoying and even distressing, but I cannot bring myself to hold that a properly-equipped and carefully-managed establishment for the relief of such pain and suffering is *per se* a noisy, noisome, or offensive business."

